# Several Feature Extraction Methods Combined with Near-Infrared Spectroscopy for Identifying the Geographical Origins of Milk

**DOI:** 10.3390/foods13111783

**Published:** 2024-06-06

**Authors:** Xiaohong Wu, Yixuan Wang, Chengyu He, Bin Wu, Tingfei Zhang, Jun Sun

**Affiliations:** 1 School of Electrical and Information Engineering, Jiangsu University, Zhenjiang 212013, China; 2222207041@stmail.ujs.edu.cn (Y.W.); 2222107003@stmail.ujs.edu.cn (C.H.); 2221907104@stmail.ujs.edu.cn (T.Z.); sun2000jun@ujs.edu.cn (J.S.); 2 High-Tech Key Laboratory of Agricultural Equipment and Intelligence of Jiangsu Province, Jiangsu University, Zhenjiang 212013, China; 3 Department of Information Engineering, Chuzhou Polytechnic, Chuzhou 239000, China

**Keywords:** milk, near-infrared spectroscopy, feature extraction, geographical origins, classification

## Abstract

Milk is a kind of dairy product with high nutritive value. Tracing the origin of milk can uphold the interests of consumers as well as the stability of the dairy market. In this study, a fuzzy direct linear discriminant analysis (FDLDA) is proposed to extract the near-infrared spectral information of milk by combining fuzzy set theory with direct linear discriminant analysis (DLDA). First, spectral data of the milk samples were collected by a portable NIR spectrometer. Then, the data were preprocessed by Savitzky–Golay (SG) and standard normal variables (SNV) to reduce noise, and the dimensionality of the spectral data was decreased by principal component analysis (PCA). Furthermore, linear discriminant analysis (LDA), DLDA, and FDLDA were employed to transform the spectral data into feature space. Finally, the k-nearest neighbor (KNN) classifier, extreme learning machine (ELM) and naïve Bayes classifier were used for classification. The results of the study showed that the classification accuracy of FDLDA was higher than DLDA when the KNN classifier was used. The highest recognition accuracy of FDLDA, DLDA, and LDA could reach 97.33%, 94.67%, and 94.67%. The classification accuracy of FDLDA was also higher than DLDA when using ELM and naïve Bayes classifiers, but the highest recognition accuracy was 88.24% and 92.00%, respectively. Therefore, the KNN classifier outperformed the ELM and naïve Bayes classifiers. This study demonstrated that combining FDLDA, DLDA, and LDA with NIR spectroscopy as an effective method for determining the origin of milk.

## 1. Introduction

Milk is a natural dairy product with excellent nutritional value and is popular with people around the world [1]. The content of nutrients in milk is related to climate, original pasture environment, animal feed, and other factors [2]. The origin of milk is an important element in the quality of milk and influences the price of the product. In Europe, there are policies such as the protected designation of origin (PDO) or protected geographical indication (PGI) to protect local production [3]. Due to the protected designation of origin (PDO) policies, products with PDO status command higher market values, which unfortunately makes them a prime target for counterfeiting, particularly through the forgery of product labels. Incorrect brand labels not only do harm to the reputation of the origin but also infringe on the legitimate rights and interests of consumers [4]. The traceability of milk origin is not only crucial for ensuring product safety and quality, but it also plays a significant role in safeguarding brands and promoting specialty products in their respective source regions. Therefore, finding a quick way to determine the geographical origin of milk is essential for milk food safety concerns and brand protection.

There are some methods for identifying the geographical origin of food products [5,6,7,8]. Among them, the use of stable isotope and elemental analysis is a common approach [9,10]. The distribution of isotopes and element contents in food items is affected by many factors, especially the location of origin [11]. Ng et al. [12] accurately differentiated the geographical origin of 307 milk samples by analyzing their isotopic fingerprints and elemental profiles. In addition, multiple chromatographic approaches have been developed to successfully detect the geographical origin of food products [13]. In conjunction with the nutritional composition and geographical features of milk, Xie et al. [14] employed high-performance liquid chromatography (HPLC), elemental analyzer isotope ratio mass spectrometry (EA-IRMS), and inductively coupled plasma mass spectrometry (ICP-MS) to establish the traceability of milk samples from Neimenggu. Moreover, the proton transfer reaction mass spectrometry (PTR-MS) technology is another effective method [15]. Although the above several methods can be applied to obtain the geographical information of food, they are often complex, expensive, destructive, and unsuitable for widespread use.

NIR spectroscopy is an emerging non-destructive analysis technology with the advantages of being fast, simple, and low-cost, and it has been widely applied for the analysis and classification of food [16,17,18]. Previous studies provide numerous examples where NIR spectroscopy has been utilized to facilitate both the classification of milk and the traceability of its origin. Infrared light measures the vibrations of molecules. Each functional group or structural feature of a molecule has a unique vibrational frequency [19]. When the effects of all the different functional groups are put together, a unique molecular “fingerprint” is formed that can be used to identify the sample [19]. Therefore, infrared spectroscopy can determine the composition of milk very quickly for qualitative analysis of milk.

Employing a synergistic approach, Zhang et al. [20] swiftly distinguished adulterated milk samples, which contained various pseudo-proteins and thickeners, from authentic raw milk samples. This achievement was made possible through the integration of near-infrared (NIR) spectroscopy with an advanced support vector machine (SVM) and an enhanced, non-linear simplified k-nearest neighbors (KNN) identification model. Diaz-Olivares et al. developed an on-farm online milk composition analysis system that employed NIR spectroscopy to analyze and model the composition of milk [21]. Pereira et al. successfully used NIR spectroscopy and the PLS algorithm to classify cow milk, goat milk, and mixed milk, demonstrating that NIR spectroscopy was an excellent method for the analysis of adulteration in milk [22]. Coppa et al. utilized NIR spectroscopy to acquire the feature information of milk samples to accurately distinguish pastured milk from non-pastured milk and trace their geographical origin [23]. Yin used infrared spectroscopy to test milk from four origins and developed a regression model that not only allowed for a good determination of the origin of the milk but also allowed for the detection of adulterated milk with different concentrations of urea and glucose [24].

Commercial spectroscopic equipment is often bulky and expensive, and can usually only be used in a laboratory environment, making it difficult to meet the requirement for fast, accurate, and real-time analysis in some conditions [25]. In recent years, with the advancement of science and technology, as well as the growing demand for practical applications, portable NIR spectrometers have been widely used for the analysis of samples [26]. The portable NIR spectrometers have been made more convenient for their small size, but their spectral resolution is lower than that of bulky commercial NIR spectrometers, and mathematical modeling is necessary to alleviate this weakness. The NIR spectral data of milk recorded by the portable NIR spectrometer are biased, and there are some overlapped data points between different categories of spectral data, which will have a significant impact on the model’s classification accuracy [27]. The feature extraction approach can extract meaningful information from the obtained spectral data to increase the model’s classification accuracy.

When dealing with high-dimensional data, the classical linear discriminant analysis (LDA) algorithm has several drawbacks [28]. For example, if the dimensionality of data is much greater than the number of samples, this can lead to the singularity of scattering matrices, making it difficult to find the optimal projection direction. This does not satisfy the requirement of the LDA algorithm for the within-class scattering matrix. Direct linear discriminant analysis (DLDA) is an improved dimensionality reduction technique, which extends the application of the classical LDA algorithm in the case of small sample data [29,30]. The DLDA algorithm is implemented by diagonalizing the between-class scattering matrix $S_{b}$ and the within-class scattering matrix $S_{w}$ at the same time. It discards the null space of the between-class scattering matrix $S_{b}$, but preserves the null space of the within-class scattering matrix $S_{w}$ that contains the most discriminant information. Generally, compared with LDA, the discriminant vectors of DLDA often have less redundant information and can achieve better classification results. However, DLDA is still unable to extract useful information from the overlapped NIR spectra. The theory of fuzzy set is an effective method to cope with unclear data in the field of pattern recognition. Patel et al. used the fuzzy k-nearest neighbor method to classify data instances into distinct groups. By considering both the nearest neighbors and their distances from the instances, this method effectively minimized the risk of classification errors [31]. Wang brought two fuzzy notions into LDA and successfully built a fuzzy face recognition model by investigating the structure of each layer of the two types of fuzzy systems [32]. The core thought of fuzzy set theory is to search for a parameter that measures the extent to which a sample belongs to a particular category, and this parameter is known as fuzzy membership. By recognizing the objective existence of fuzzy items, fuzzy set theory adeptly addresses the challenges posed by uncertain concepts This makes it particularly useful for extracting features from overlapped spectral data [27].

This study prefers qualitative analyses of milk from different origins. The aim is to differentiate between milk from different origins using a method that is as quick, simple, low-cost, and non-destructive as possible, whereas conducting laboratory experiments would not only take a long time and cost but also cause damage to the milk samples, which is not in line with the fundamental aim of this study. Therefore, no laboratory measurements were required in this study.

The purpose of this study was to create a model that could rapidly and reliably identify the geographical origin of milk by combining fuzzy set theory with feature extraction methods. The research steps were as follows: (1) A portable NIR spectrometer was employed to collect spectral data from milk samples. (2) Several data processing algorithms were integrated to deal with the spectra. Savitzky–Golay (SG) and standard normal variables (SNV) were conducted to remove noise, and principal component analysis (PCA) was applied for dimension reduction. Three feature extraction methods were used to separate the spectral data. (3) The k-nearest neighbor (KNN) classifier, extreme learning machine (ELM), and naïve Bayes classifiers were used to classify the data after feature extraction.

## 2. Materials and Experiments

### 2.1. Internal Content Difference between Milks

The nutrient content in milk is related to the place of origin [33]. Ningxia had favorable temperatures all year round, which provided sufficient feed resources for dairy farming [34]. While Henan dairy cattle feed mainly relied on straw and natural pasture, there was a serious shortage of high-quality feed. Table 1 summarizes the nutrient content of milk from different regions.

The origin of milk could be distinguished by a stable isotope [35]. The $\delta^{13}C$ of milk was mainly related to the composition of the cow’s food [36]. $\delta^{15}N$ mainly reflected the soil conditions of the environment in which the cow lived and the number of legumes in the ration. $\delta^{18}O$ and $\delta^{2}H$ were closely related to the environmental factors of the local water source, air, and climatic conditions. Table 2 shows the results of stable isotope ratio measurements of milk samples from different regions. 

Differences in mineral elements also provided important support for identifying the geographical origin of milk [37]. Table 3 shows the contents and significant differences of different mineral elements in milk samples. These differences were caused by the different feeding methods of the cows. The trace elements were present at low levels in living organisms and depended mainly on soil and geological features.

Table 3 shows that there are differences in the type and content of components in milk from different origins. Therefore, the use of NIR spectroscopy and feature extraction techniques to classify the origin of milk is scientifically sound and also highly feasible.

### 2.2. Sample Preparation

The five different commercial milks used in the experiment were purchased from the local supermarket in Zhenjiang, China. They came from five different provinces: Heilongjiang, Hebei, Henan, Ningxia, and Neimenggu, which are the main dairy production bases in China. Samples needed to be selected with attention to the exact origin information, date of manufacture, and nutritional content labeled on the box. To ensure the objectivity of the experiment, all five different commercial milks were purchased on the same day and the production date of them needed to be as close as possible. To test the classification performance of the model, semi-skimmed milk was selected for milk from Hebei, whole milk was selected for milk from the other four origins. Ultra-high temperature sterilized milk was selected for all milk samples to facilitate subsequent experiments and storage. For a total of 300 samples, 60 of each type of milk were obtained. All milk samples were kept in the laboratory at a temperature of 25 ± 1 °C to preserve the milk’s quality.

### 2.3. Instruments and Software

A reflectance handheld NIR spectrometer NIR-M-R2 (Shenzhen Pynect Science and Technology Co., Ltd., Shenzhen, China) was used in this study. The spectrometer captured spectra in the wavelength range of 900–1700 nm with an optical resolution of 10 nm, a signal-to-noise ratio of 6000:1, and a slit size of 1.8 mm × 0.025 mm. It also had a temperature and humidity sensor for displaying temperature and humidity measurements in the module.

All algorithms mentioned were programmed and executed in MATLAB (The Mathworks Inc., Natick, MA, USA) 2019b on a personal computer with the operating system Microsoft Windows 10.

### 2.4. Spectra Collection

Firstly, the spectral dimension in this experiment was set to 228 and the spectrometer was turned on and preheated for half an hour. After that, 5 mL of milk was taken from each milk sample by using a pipette and placed in a 10 mL beaker. During the scanning process, the beaker needed to be kept at a constant distance from the portable NIR spectrometer to ensure the stability of the experiment. The milk NIR spectra recorded in this experiment had a wavelength range of 5882 ${cm}^{-1}$–11,111 ${cm}^{-1}$ and a data dimension of 228. Each milk sample was used three times, and the average value of the three spectral data served as the final NIR spectral data.

### 2.5. NIR Spectra Preprocessing

When collecting spectral data, due to the impacts of measurement errors and optical scattering, the raw spectral data were mixed with a large amount of noise. To improve the accuracy of the classification, the raw spectra must be preprocessed to remove the influence of noise and scattering on the spectral data [38].

Multiplying scattering correction (MSC), standard normal variables (SNV), Savitzky–Golay (SG) and mean centering (MC) were used in this study. For these four data preprocessing methods, each serves a distinct purpose [39]. MSC is primarily employed to minimize the scattering effect induced by irregular particle distribution and particle size. SNV is applied to remove the impact of variations in solid particle size, apparent scattering, and optical paths. SG can remove scattering phenomena, increase spectral data smoothness, and lower the impact of diffuse reflections and noise interference. The MC could magnify weak signals.

### 2.6. Division of Training Set and Test Set

A total of 300 spectral data were obtained. The data were divided into a training set and a test set in a ratio of 3:1, with 45 training samples and 15 test samples for each milk sample. This means that there were 225 samples in the training set and 75 samples in the test set.

### 2.7. Principal Component Analysis

PCA is the most commonly used dimensionality reduction algorithm in the field of machine learning. PCA can map the features of spectral data into a few important features while discarding unnecessary features. PCA is always utilized to reduce data dimension and remove the redundant information in the spectral data. It reduces the dimensionality of data and maintains the most important information of the high-dimensional data by orthogonally transforming a large number of significant variables into a few uncorrelated groups of variables. PCA can remove noise and unnecessary features, and this is advantageous to improve data processing speed and classification accuracy. In practical application, the amount of the chosen principal components has a significant impact on the classification results [40,41]. Selecting appropriate principal components and retaining the relevant important features are conducive to the improvement of classification accuracy.

## 3. Feature Extraction Methods

NIR spectroscopy suffers from defects such as wide spectral band, severe overlap, and complex information resolution, so it can only be used as an indirect measurement method. Therefore, appropriate metrological models need to be established to qualitatively analyze the samples. Feature extraction converts the target sample set from a high-dimensional feature space to a low-dimensional feature space, making the projected sample easy to distinguish.

### 3.1. Linear Discriminant Analysis

The LDA algorithm is a supervised feature extraction method. Using a given training set of samples, the sample points are projected onto a straight line. To obtain the minimum distances within a class and the maximum distances between classes, the projection points of samples belonging to the same class need to be as close together as feasible, while those belonging to different classes need to be as far apart. However, when the dimension of the sample data is larger than the number of sample data, it will result in the required scattering matrix being a singular matrix, which prevents the subsequent calculations. Therefore, an improvement of LDA is needed.

### 3.2. Direct Linear Discriminant Analysis

Direct linear discriminant analysis (DLDA) is an improved feature extraction method that extends the application of the classical LDA algorithm in the context of small sample setting (SSS). At the heart of DLDA is the idea of simultaneous diagonalization, which tries to find a matrix that can directly diagonalize the scattering matrix [42]. DLDA is computed in two phases: the first phase computes the conversion matrix to transform the training samples into the range space of the scattering matrix; the second phase further reduces the dimensionality of the samples through some conditioning matrices [30]. Because the NIR spectral data of milk have high dimensions, DLDA can be utilized to analyze the data and extract useful information.

The specific steps of the DLDA algorithm are as follows (input: data matrix X; output: transformation matrix $W_{DLDA}$) [42]:

- Step 1. Calculate matrix $S_{w}$ and $S_{b}$;
- Step 2. Apply the eigendecomposition on $S_{b}$ to get Y and $D_{b}$, then $Z\leftarrow YD_{b}^{-1/2}$;
- Step 3. Compute the eigenvalues and eigenvectors of $Z^{T}S_{w}Z$ to get $D_{w}$ and U;
- Step 4. Obtain the transformation matrix: $W_{DLDA}=D_{w}^{-1/2}U^{T}Z^{T}$.

In step 1, the within-class scattering matrix $S_{w}$ and the between-class scattering matrix $S_{b}$ are calculated as follows:

(1) $$S_{w}=\sum_{i=1}^{c}\sum_{k=1}^{n_{i}}({\bar{x}}_{i}-x_{k})({\bar{x}}_{i}-x_{k}{)}^{T}$$

(2) $$S_{b}=\sum_{i=1}^{c}n_{i}({\bar{x}}_{i}-\bar{x})({\bar{x}}_{i}-\bar{x}{)}^{T}$$

In the above equations, c represents the number of sample categories; $n_{i}$ represents the number of samples in the *i*th category; $\bar{x}$ represents the average value of the total sample; and ${\bar{x}}_{i}$ represents the average value of the *i*th category.

### 3.3. Fuzzy Direct Linear Discriminant Analysis

When faced with complex datasets, DLDA sometimes fails to extract feature information from data, and it is especially difficult to solve the problem of overlapped data. Fuzzy set theory is an effective method for analyzing uncertain data.

Before using the fuzzy theory, the initial cluster centers and fuzzy membership values need to be calculated first. The initial cluster center of fuzzy direct linear discriminant analysis (FDLDA) was the mean value of each kind of training sample. The parameters were set as follows: the fuzzy weight coefficient m=3.5, the number of sample category c=5, and the five initial cluster centers constituted the matrix $V^{\left(0\right)}$ [27]:

(3) $$V^{\left(0\right)}=\left[\begin{array}{c} v_{1}^{\left(0\right)}\\ v_{2}^{\left(0\right)}\\ v_{3}^{\left(0\right)}\\ v_{4}^{\left(0\right)}\\ v_{5}^{\left(0\right)} \end{array}\right]=\left[\begin{array}{ccccc} -0.5798 & -0.0148 & 0.0123 & 0.1106 & 0.1268\\ -0.1045 & 0.1909 & 0.0445 & -0.0329 & -0.0006\\ 0.1359 & 0.3192 & -0.0618 & -0.0250 & -0.0645\\ 0.8331 & -0.1365 & -0.0003 & 0.0707 & 0.0170\\ -0.2921 & -0.3019 & -0.0264 & -0.0652 & -0.1108 \end{array}\right]$$

Classical set theory suggests that there are only two states of the properties of objects: belonging or not belonging, i.e., 1 or 0. Zadeh first proposed fuzzy set theory [43]. Fuzzy set theory measures the probability of a sample belonging to a category through the concept of fuzzy membership value. The fuzzy membership value varies between 0 and 1, with the closer to 0 indicating that a sample does not belong to that state, and the closer to 1 indicating that it is more likely to belong to that state [44]. By combining DLDA with the fuzzy set theory, a fuzzy direct linear discriminant analysis (FDLDA) algorithm is proposed. FDLDA will have an advantage over DLDA when dealing with overlapped data.

The operation steps of FDLDA are as follows (input: data matrix X; output: transformation matrix $W_{FDLDA})$:

- Step 1. Calculate matrix $S_{fw}$ and $S_{fb}$;
- Step 2. Apply the eigendecomposition on $S_{fb}$ to get $Y_{f}$ and $D_{fb}$, then $Z_{f}\leftarrow Y_{f}D_{fb}^{-1/2}$;
- Step 3. Compute the eigenvalues and eigenvectors of ${Z_{f}}^{T}S_{fw}Z_{f}$ to get $D_{fw}$ and $U_{f}$;
- Step 4. Obtain the transformation matrix: $W_{FDLDA}=D_{fw}^{-1/2}{U_{f}}^{T}{Z_{f}}^{T}$.

In step 1, the within-class scattering matrix $S_{fw}$ and the between-class scattering matrix $S_{fb}$ are calculated as follows:

(4) $$S_{fw}=\sum_{j=1}^{c}\sum_{i=1}^{n}U_{ij}^{m}(x_{i}-{\bar{x}}_{j})(x_{i}-{\bar{x}}_{j}{)}^{T}$$

(5) $$S_{fb}=\sum_{j=1}^{c}\sum_{i=1}^{n}U_{ij}^{m}({\bar{x}}_{j}-\bar{x})(x_{j}-\bar{x}{)}^{T}$$

Here, c represents the number of categories; n represents the number of training samples; $U_{ij}$ represents the degree to which the *i*th sample data $x_{i}$ belongs to category *j*, and the value of $U_{ij}$ varies from 0 to 1; $\bar{x}$ represents the average value of the training samples; and ${\bar{x}}_{j}$ represents the average value of the *j*th category.

## 4. Classifiers

### 4.1. K-Nearest Neighbor (KNN) Classifier

KNN is a type of supervised classifier. It does not have a learning process in the general sense but rather uses the training data to partition the feature vector space, and the result of the partitioning is used as the final algorithmic model [45]. In order to categorize test samples, it first scans the training set in search of a training sample that is the most similar to the test sample. It then votes based on the class of this sample to establish the kind of test samples [46]. The classification accuracy of KNN is influenced by the parameters of *K*, distance metric, and the number of samples. To avoid identical votes in the poll, the parameter *K* is always set to an odd integer in the algorithm [47].

### 4.2. Extreme Learning Machine (ELM)

The extreme learning machine (ELM) stands as a prevalent classifier in the realm of pattern recognition [48]. The ELM can randomly initialize the input weights and biases, and once the weights and biases have been randomly determined, the output matrix of the hidden layer is uniquely determined [49]. This distinctive training process allows for the efficient learning of complex non-linear mapping relationships, endowing ELM with speed and versatility in model training [48].

### 4.3. Naïve Bayes Classifier

The naïve Bayes classifier is a statistical classification algorithm based on Bayes’ theorem. Throughout the training phase, the algorithm constructs the model through the estimation of both prior probabilities and conditional probabilities [50]. In the classification stage, it computes the posterior probability for each category and subsequently designates the category possessing the highest posterior probability as the classification outcome for the given sample.

## 5. Results and Discussion

### 5.1. Spectral Analysis

**Figure 1:**
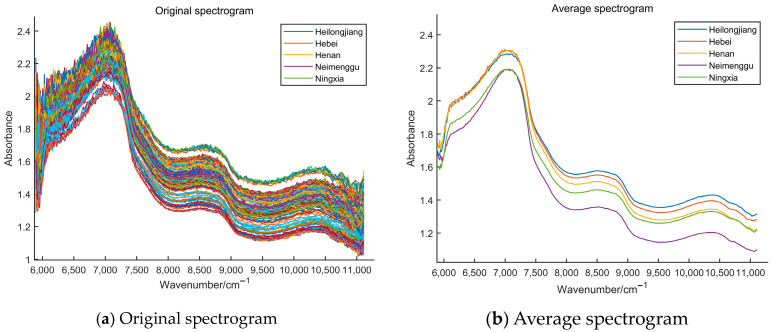
The spectra of milk samples from 5 different provinces (**a**) original spectrogram; (**b**) average spec-trogram.

In this study, the NIR spectra from five different types of milk were obtained. Figure 1 shows the spectral data of milk samples from five different provinces. There is an absorption peak in the NIR spectral data of milk at 7112 ${cm}^{-1}$. There is an overlap in the spectra of milk from Neimenggu and Ningxia at 7112 ${cm}^{-1}$, as well as an overlap in the spectra of milk from Heilongjiang, Hebei, and Henan. The absorption peaks here are shifted by the absorption bands due to the difference in lactose content [51]. It can be inferred that there was a difference in lactose content between milk from Neimenggu and Ningxia, and milk from Heilongjiang, Hebei, and Henan. The fluctuation at 10,417 ${cm}^{-1}$ is related to the O-H bond content in water [52]. It can be inferred that the content of water in milk from Ningxia and Henan is similar, while the content of water in milk from the other three origins is more different. The fluctuations at 6579 ${cm}^{-1}$, 6849 ${cm}^{-1}$, and 8833 ${cm}^{-1}$ are affected by the N-H bond in the protein [53]. It can be inferred that the protein content of milk from different origins varies greatly. The first-order frequency doubling of the stretching vibration of the C-H bond in fat is related to the fluctuation at 6250 ${cm}^{-1}$ [54]. It can be inferred that the fat content of milk from Heilongjiang, Hebei, and Henan is similar and very different from that of milk from Neimenggu and Ningxia. Meanwhile, other components in milk may also contain C-H, N-H, and O-H bonds, which can also lead to fluctuations in the spectral curves. So, there are differences in the components and content of milk from the five origins. In general, there are differences in the type and number of chemical bonds in commercial milk from five different origins. Therefore, we can distinguish milk from different origins based on fluctuations in the spectral curves.

### 5.2. Results of Different Spectral Preprocessing

**Figure 2:**
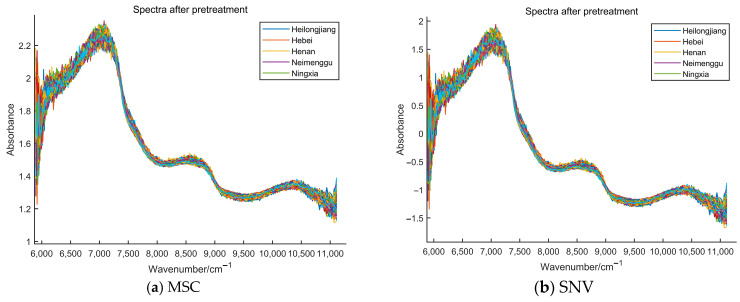

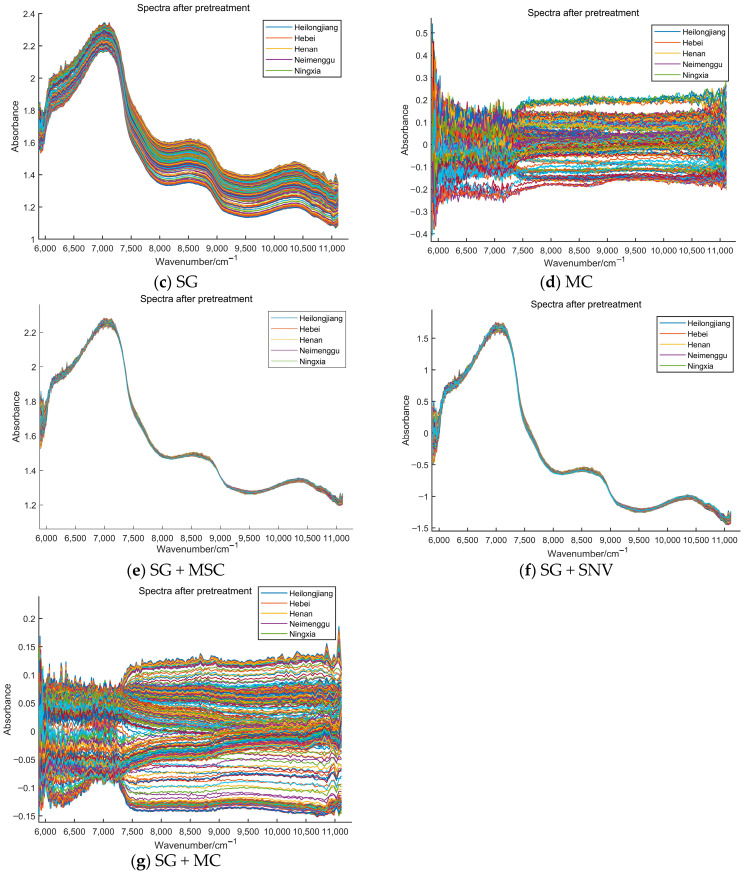
NIR spectra of milk samples under different pretreatment methods: (**a**) multiplying scattering correction (MSC); (**b**) standard normal variables (SNV); (**c**) Savitzky–Golay (SG); (**d**) mean centering (MC); (**e**) Savitzky–Golay and multiplying scattering correction (SG + MSC); (**f**) Savitzky–Golay and standard normal variables (SG + SNV); (**g**) Savitzky–Golay and mean centering (SG + MC).

Figure 2 shows the NIR spectra of milk after some pretreatment procedures. MSC, SNV, SG, MC, SG + MSC, SG + SNV, and SG + MC were the data preprocessing methods used in this study. A comparison of (a), (b), (c), and (d) in Figure 2 showed that the spectrogram acquired by the SG pretreatment approach was smoother than those obtained by the other approaches. To see if better results could be obtained, the acquired data were processed using SG in conjunction with other preprocessing methods (e.g., SG + MSC, SG + SNV, and SG + MC). The spectrograms preprocessed by SG + MSC, SG + SNV, and SG + MC are shown in (e), (f), and (g).

Table 4 demonstrates the classification accuracy of the model after using different preprocessing methods. It could be seen that the model’s classification accuracies were low when the preprocessing methods were MSC, SNV, and MC, indicating that the spectra by the preprocessing methods in Figure 2a,b,d with high noise and distortion. The classification accuracies under the SG, SG + MSC, SG + SNV, and SG + MC preprocessing methods were high, and they were corroborated by the comparative smoothing of the spectra in Figure 2c,e,f,g with no significant noise. Table 4 also indicated that the classification accuracies of the combined two preprocessing methods were higher than those of only one preprocessing method.

### 5.3. PCA for Dimensionality Reduction

Principal component analysis (PCA) was employed to reduce the dimensionality of the NIR spectral data after the preprocessing of the milk spectral data. The number of major components was often determined by the cumulative contribution rate as a criterion. Table 5 illustrates the classification accuracies of LDA, DLDA, and FDLDA with different numbers of principal components. LDA had the maximum classification accuracy with 94.67% when the number of principal components (NPC) was set as seven. When the NPC was set as five, DLDA and FDLDA had the highest classification accuracies with 94.67% and 97.33%, respectively. However, when the NPC was set as 11, LDA and DLDA had relatively low classification accuracies. As a result, the NPC was set as five in this study.

When there were five principal components in this study, the total contribution of the first five principal components reached 99.99%, and they could roughly capture the feature information of all the data. The eigenvalues were as follows: $\lambda_{1}=66.3279$; $\lambda_{2}=15.2091$; $\lambda_{3}=3.5933$; $\lambda_{4}=2.8142$; $\lambda_{5}=1.8735$; $\lambda_{6}=1.1185$; $\lambda_{7}=0.8914$; $\lambda_{8}=0.7189$; $\lambda_{9}=0.6504$; $\lambda_{10}=0.5339$; $\lambda_{11}=0.3589$.

**Figure 3:**
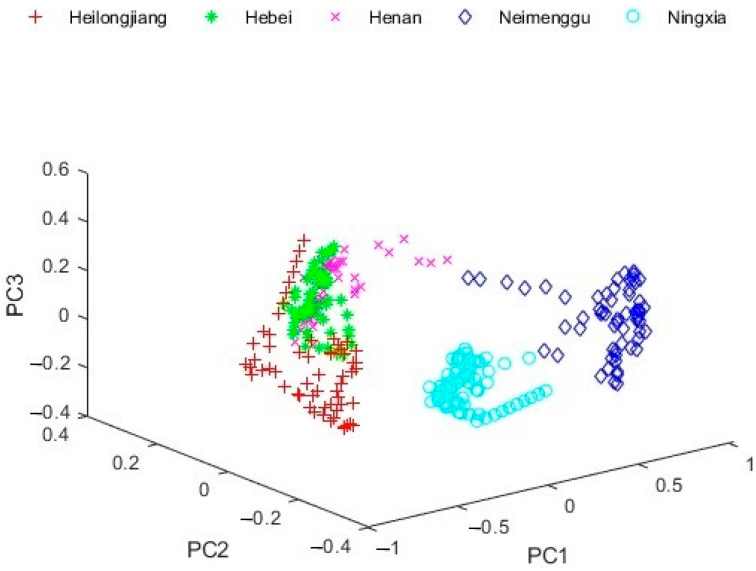
The data distribution after PCA.

By dimensionality reduction of the spectral data through PCA, the data point distribution is shown in Figure 3. It could be seen that there was a large overlap of milk sample data from Heilongjiang, Henan, and Hebei, which increased the difficulty of the subsequent classification task.

### 5.4. Establishment of Discriminant Model

#### 5.4.1. Feature Extraction with DLDA

Since the PCA dimensionality reduction process set the NPCs to five, this meant that PCA reduced the original 228-dimensional data to five dimensions. For feature extraction using DLDA, the transformation matrix further reduced the dimensionality of the sample data to four dimensions (number of categories − 1). This process generated the four best discriminant vectors (DV1, DV2, DV3, and DV4).

**Figure 4:**
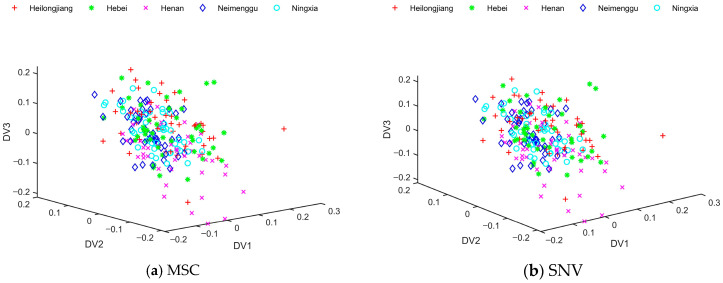

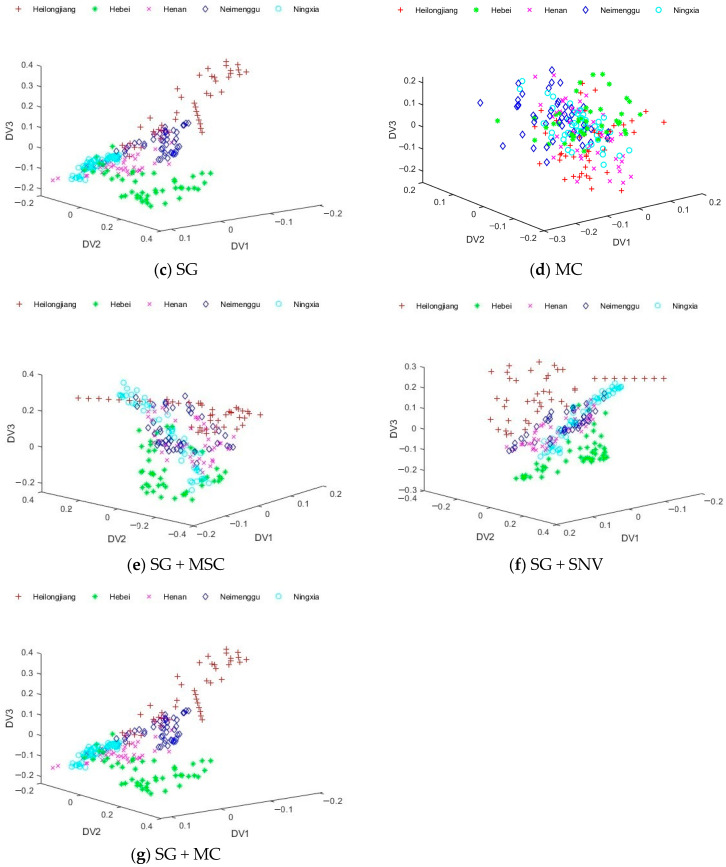
The distribution of training samples after direct linear discriminant analysis (DLDA) under different preprocessing methods: (**a**) multiplying scattering correction (MSC); (**b**) standard normal variables (SNV); (**c**) Savitzky–Golay (SG); (**d**) mean centering (MC); (**e**) Savitzky–Golay and multiplying scattering correction (SG + MSC); (**f**) Savitzky–Golay and standard normal variables (SG + SNV); (**g**) Savitzky–Golay and mean centering (SG + MC). (There are 45 training samples for each type of commercial milk, totaling 225 training samples for the 5 commercial kinds of milk.).

As can be seen from Figure 4, the distribution of sample data after DLDA feature extraction was chaotic. Most of the milk samples from Heilongjiang and Hebei were able to be separated when pretreated with SG, SG + SNV, and SG + MC. However, there was a large overlap in milk samples from Henan, Neimenggu, and Ningxia. DLDA did not distinguish milk samples from different origins very well. In order to deal with these overlapped data, this study needed to introduce the fuzzy theory and develop a new feature extraction algorithm.

#### 5.4.2. Feature Extraction with FDLDA

**Figure 5:**
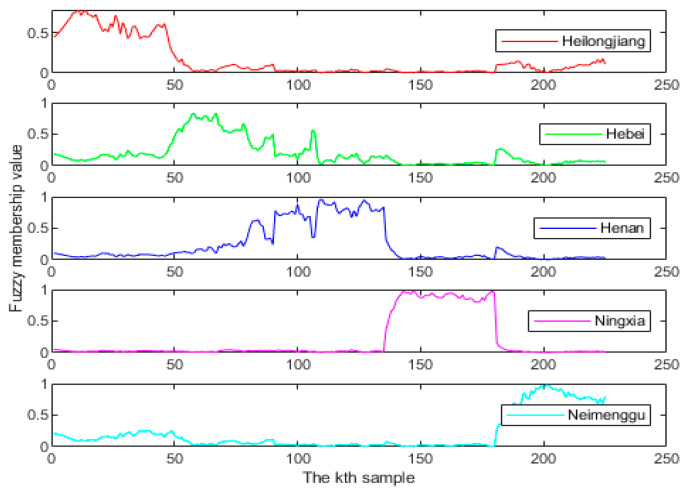
Fuzzy membership values of milk samples.

As shown in Figure 5. The horizontal and vertical axes indicated the number of samples and the fuzzy membership value, respectively. The fuzzy membership value in Figure 5 was calculated using Euclidean distance, which indicated the probability that each of the 225 samples in the training set belonged to each category. When the fuzzy membership value was closer to 1, it meant that the probability of the sample belonging to that category was higher. Similarly, if the fuzzy membership value was closer to 0, it meant that the probability of the sample belonging to that category was less. For example, the fourth subplot of Figure 5 represented the probability of being from Ningxia among the 225 milk training samples. It could be seen that the fuzzy membership value of the 136th–180th samples was very close to 1, while the rest of the samples had a fuzzy membership value of basically 0. This indicated that the 136th–180th milk samples were more likely to be from Ningxia, while the rest of the samples were more likely to be from the other four provinces.

**Figure 6:**
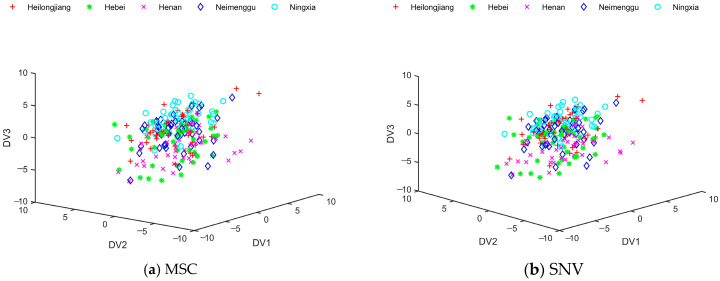

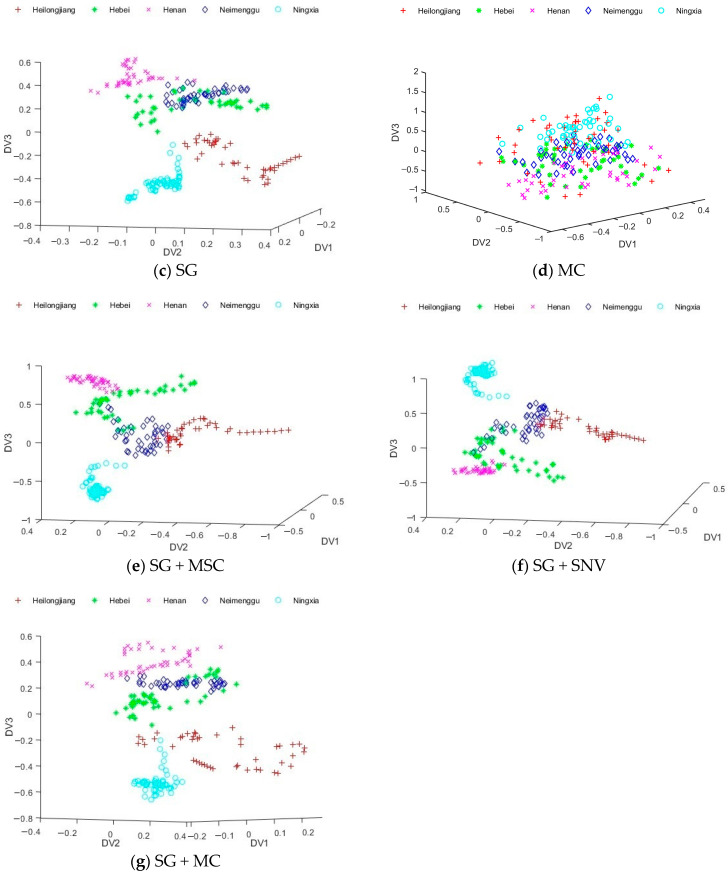
The distribution of training samples after fuzzy direct linear discriminant analysis (FDLDA) under different preprocessing methods: (**a**) multiplying scattering correction (MSC); (**b**) standard normal variables (SNV); (**c**) Savitzky–Golay (SG); (**d**) mean centering (MC); (**e**) Savitzky–Golay and multiplying scattering correction (SG + MSC); (**f**) Savitzky–Golay and standard normal variables (SG + SNV); (**g**) Savitzky–Golay and mean centering (SG + MC). (There are 45 training samples for each type of commercial milk, totaling 225 training samples for the 5 commercial kinds of milk.).

Figure 6 shows the distribution of results for extracting discrimination information from milk samples using FDLDA as a feature extraction technique. The figure showed a good separation of data points for the five types of samples. The study also found that the accuracies were improved by combining two preprocessing methods, such as SG + MSC, SG + SNV, and SG + MC, compared to using the SG algorithm alone. As shown in Figure 4 and Figure 6, compared with DLDA, the distribution boundaries between different types of milk data processed by FDLDA were much clearer, which considerably solved the problem of data overlapping. The classification accuracy of FDLDA achieved 97.33% in the classification of milk samples from five distinct geographical origins.

Combined with Table 4, it could also be seen that the classification accuracies of the FDLDA model were higher than those of the DLDA model when using SG, SG + SNV, SG + MSC, and SG + MC as preprocessing methods. In particular, it could be inferred that the ability of FDLDA to separate overlapped data was better than that of DLDA. Therefore, combining FDLDA with NIR spectroscopy could effectively determine the geographical origin of milk.

**Figure 7:**
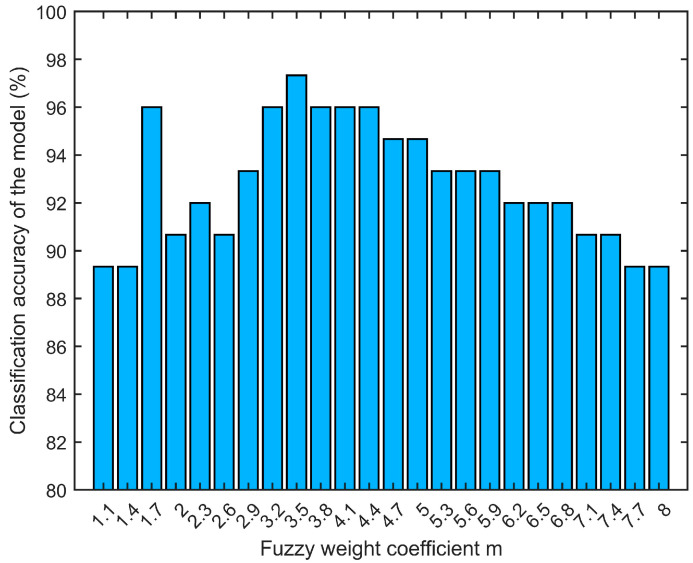
Classification results of FDLDA with different values of weight coefficient m.

The fuzzy weight coefficient m had a significant impact on the final results when FDLDA was applied for feature extraction (SG + SNV was the preprocessing algorithm, and the classifier was KNN with parameter *K* = 7). It was difficult for FDLDA to separate the data if m was too small, and the fuzzy membership values of different kinds of data would become ambiguous if m was too large. Figure 7 shows the FDLDA model’s classification accuracies for different fuzzy weight coefficient *m* values. The classification accuracy increased gradually as m increased. When the fuzzy weight coefficient m was 3.5, the classification accuracy reached its peak at 97.33%.

### 5.5. Classification Results

#### 5.5.1. Classification by KNN Classifier

This research integrated several data preprocessing methods and feature extraction algorithms to analyze the spectral data of milk samples and then applied a KNN classifier to achieve effective discrimination of the geographical source of milk. The choice of *K* value had a significant impact on the performance. Table 6 demonstrates the classification accuracies of LDA, DLDA, and FDLDA with different *K* values (the preprocessing method was SG + SNV; the number of principal components was 5; the fuzzy weight coefficient m=3.5). As seen in Table 6, with the same *K* value, the classification accuracy of FDLDA was higher than LDA and DLDA. Furthermore, FDLDA achieved the highest recognition accuracy 97.33% when *K* = 7. The results presented above suggest that FDLDA was a more suitable algorithm for feature extraction when processing NIR spectra of milk samples, particularly in cases where the data exhibits some degree of overlap.

#### 5.5.2. Classification by ELM

Subsequently, the classification performance of the FDLDA model was validated using an extreme learning machine (ELM). The number of hidden layer neurons was a crucial hyperparameter that directly impacted the model’s performance and generalization ability. In this study, the optimal number of hidden layer neurons was selected from the range of 1 to 20. The optimal numbers of hidden layer neurons for the LDA, DLDA, and FDLDA models were determined to be 7, 16, and 10, respectively. Due to the stochastic nature of ELM results, the average of 50 experimental results was taken as the final result. Table 7 displays the classification accuracies of the models under different preprocessing methods (the value of the fuzzy weight coefficient m was 3). The findings indicated that when employing SG, SG + MC, and SG + SNV as preprocessing methods, the LDA, DLDA, and FDLDA models achieved the highest classification accuracies. Notably, the FDLDA model exhibited superior classification accuracy compared to both the LDA and DLDA models.

#### 5.5.3. Classification by Naïve Bayes Classifier

In the end, the classification performance of the FDLDA model was assessed by employing a naïve Bayes classifier. Table 8 displays the classification accuracy using the naïve Bayes classifier under different preprocessing methods, with the weight coefficient m set to 2. The highest classification accuracies of LDA, DLDA, and FDLDA models were more than 90%, specifically, 93.33%, 90.67%, and 92.00%, respectively. It was noteworthy that the FDLDA model exhibited higher classification accuracy compared to the DLDA model, indicating that FDLDA was better suited for handling overlapped data than DLDA.

### 5.6. Comparative Analysis

The model FDLDA-KNN proposed in this study achieved a classification accuracy of 97.33% for milk test samples. Marijan et al. successfully differentiated between sheep’s milk from various geographical origins by accurately determining the stable isotope ratios and elemental composition of the primary bio-elements, achieving an impressive accuracy rate of 97.00% [55]. Liu et al. successfully classified goat milk from three pastures in China. This accomplishment was realized through the precise determination of lipid molecules present in goat milk samples from these regions, resulting in an impeccable classification accuracy of 100% [56]. Ng et al. determined the stable isotope signature and elemental concentration of milk to differentiate between milk from Australia, New Zealand, Thailand, and the USA, achieving a classification accuracy of 94.7% [12]. Magdas et al. studied the isotopic and elemental composition of raw milk from Transylvania and successfully differentiated raw milk from three pastures with 90.9% classification accuracy [57].

The comparison shows that most of the origin traceability for milk was performed by determining its composition. Although achieving high accuracy, this method is complex, time-consuming, and lossy, making it difficult to commercialize on a large scale. In contrast, the origin traceability model proposed in this study is fast and non-destructive while still achieving high classification accuracy. This provides a reference for future research.

## 6. Conclusions

In this study, the NIR spectral data of the milk were acquired by a portable NIR spectrometer. The data were preprocessed by different kinds of data preprocessing methods, and then PCA was applied to reduce the dimensionality of the data. LDA, DLDA, and FDLDA were employed to obtain feature information from the NIR spectra of milk. Finally, the KNN classifier, ELM, and naïve Bayes classifier were applied to classify five types of milk samples. Experimental results indicated that the KNN classifier outperformed ELM and the naïve Bayes classifier in terms of classification accuracy. FDLDA-KNN achieved the highest classification accuracy of 97.33% for the milk test samples. This meant that the FDLDA-KNN model showed greater ability than the DLDA-KNN model to handle overlapped data, clarify data boundaries, and achieve the highest classification accuracy. In summary, the combination of FDLDA with near-infrared spectroscopy offered an effective method for identifying the geographical origin of milk.

Milk from different origins varies in terms of its composition and content, which results in different types and amounts of chemical bonds. NIR spectroscopy can detect the expansion and contraction vibration of different chemical bonds, so as long as the milks are from different origins, there will be differences in their spectra. And the algorithm proposed in this study first learns using the training data to extract discriminant information and then classifies the test data. So, this study can be generalized to a large extent to milk from other origins and it is not limited by the origins selected in this study.

At present, the integration of NIR spectroscopy technology with other technologies has become the mainstream of food origin traceability research. NIR spectroscopy has played an important role in the food industry, but the mechanism of origin traceability and the establishment of various discriminative models are still in the exploratory stage. A complete traceability system has not yet been established. This study provides a reference for the traceability of other dairy products or food in the future. The research on the origin traceability of meat products is less, and the geographical origin of each field needs to be studied from multiple angles in the future. It is also possible to think about the combination of spectroscopic techniques, chemometrics methods, and other techniques (e.g., radio frequency identification) to build a complete food traceability system.

## Figures and Tables

**Table 1:** Comparison of the nutrient content of milk in different regions.

Origin	Fat (%)	Protein (%)	Lactose (%)	Total Solids (%)
Henan	3.18 ± 1.16 ^c^	3.51 ± 0.47 ^b^	4.95 ± 0.23 ^b^	12.14 ± 1.38 ^c^
Neimenggu	3.44 ± 0.92 ^b^	3.43 ± 0.43 ^b^	5.09 ± 0.23 ^b^	12.45 ± 1.21 ^b^
Ningxia	3.52 ± 0.85 ^b^	3.67 ± 0.41 ^a^	5.06 ± 0.24 ^a^	12.78 ± 0.18 ^ab^

Values in the table are mean ± standard deviation, different letters labelled on the shoulder of peer data indicate significant differences (*p* < 0.05), and having the same letter indicates non-significant differences (*p* > 0.05).

**Table 2:** Results of stable isotope ratio measurements of milk samples from different regions.

Origin	$\delta^{13}C$	$\delta^{15}N$	$\delta^{18}O$	$\delta^{2}H$
Heilongjiang	−18.389 ± 0.252 a	2.976 ± 0.193 b	−158.990 ± 15.181 c	8.858 ± 1.807 bc
Hebei	−18.456 ± 0.139 ab	3.309 ± 0.075 a	−144.723 ± 2.129 b	9.880 ± 0.297 b
Neimenggu	−19.138 ± 0.429 c	3.501 ± 0.139 a	−147.340 ± 11.573 bc	10.036 ± 0.350 b

Values in the table are mean ± standard deviation, different letters labelled on the shoulder of peer data indicate significant differences (*p* < 0.05), and having the same letter indicates non-significant differences (*p* > 0.05).

**Table 3:** The mineral content of milk from different origins.

Element	Hebei	Neimenggu	Ningxia
Na (mg/kg)	2046 ± 429 ^a^	2695 ± 422 ^b^	3358 ± 511 ^c^
Mg (mg/kg)	879 ± 129 ^a^	1232 ± 134 ^b^	1161 ± 363 ^c^
K (mg/kg)	12,899 ± 2773 ^a^	156,616 ± 2179 ^b^	22,872 ± 5023 ^c^
Ca (mg/kg)	8113 ± 1073 ^b^	3843 ± 487 ^a^	12,042 ± 3399 ^c^
Ti (mg/kg)	40.5 ± 7.80 ^a^	64.8 ± 7.20 ^d^	56.5 ± 9.39 ^b^
Cr (mg/kg)	44.0 ± 7.71 ^a^	176 ± 29.5 ^d^	88.3 ± 18.3 ^b^
Mn (mg/kg)	460 ± 130 ^a^	1055 ± 216 ^c^	751 ± 256 ^b^
Fe (mg/kg)	62.9 ± 9.63 ^a^	70.2 ± 7.66 ^b^	84.6 ± 13.3 ^c^
Ni (mg/kg)	348 ± 53.3 ^a^	549 ± 82.0 ^bc^	585 ± 130 ^c^
Zn (mg/kg)	34.9 ± 7.06 ^a^	75.3 ± 12.0 ^c^	34.3 ± 7.84 ^a^
Sr (mg/kg)	4.10 ± 0.935 ^a^	11.5 ± 2.16 ^c^	7.96 ± 1.49 ^b^

Values in the table are mean ± standard deviation, different letters labelled on the shoulder of peer data indicate significant differences (*p* < 0.05), and having the same letter indicates non-significant differences (*p* > 0.05).

**Table 4:** Classification accuracy of models with different preprocessing algorithms (%).

Feature Extraction	MSC	SNV	SG	MC	SG + MSC	SG + SNV	SG + MC
LDA	30.67	32.00	94.67	64.00	92.00	90.67	82.67
DLDA	38.67	40.00	90.67	60.00	93.00	94.67	92.00
FDLDA	34.67	34.67	94.67	66.67	96.00	97.33	94.67

Abbreviation: MSC: multiplying scattering correction; SNV: standard normal variables; SG: Savitzky–Golay; MC: mean centering; LDA: linear discriminant analysis; DLDA: direct linear discriminant analysis; FDLDA: fuzzy direct linear discriminant analysis.

**Table 5:** Classification accuracy under different numbers of principal components (%).

PCs	4	5	6	7	8	9	10	11	12
LDA	88.00	90.67	90.67	94.67	94.67	92.00	90.67	80.00	76.00
DLDA	94.67	94.67	93.33	92.00	94.67	89.33	86.67	89.33	82.67
FDLDA	94.67	97.33	89.33	93.33	90.67	94.67	94.67	97.33	92.00

Abbreviation: PCs: principal components; LDA: linear discriminant analysis; DLDA: direct linear discriminant analysis; FDLDA: fuzzy direct linear discriminant analysis.

**Table 6:** Classification accuracy of the model using KNN with different *K* values (%).

*K*	1	3	5	7	9	11
LDA	85.33	88.00	93.33	90.67	88.00	90.67
DLDA	82.67	86.67	93.33	94.67	89.33	89.33
FDLDA	88.00	93.33	96.00	97.33	93.33	96.00

Abbreviation: LDA: linear discriminant analysis; DLDA: direct linear discriminant analysis; FDLDA: fuzzy direct linear discriminant analysis.

**Table 7:** Classification accuracy of the model obtained using ELM with different preprocessing methods (%).

Feature Extraction	MSC	SNV	SG	MC	SG + MSC	SG + SNV	SG + MC
LDA	53.25	52.53	85.92	65.97	83.93	82.56	84.90
DLDA	52.40	51.70	84.96	66.88	81.01	81.04	86.03
FDLDA	51.45	51.87	87.14	66.67	87.25	88.24	86.42

Abbreviation: MSC: multiplying scattering correction; SNV: standard normal variables; SG: Savitzky–Golay; MC: mean centering; LDA: linear discriminant analysis; DLDA: direct linear discriminant analysis; FDLDA: fuzzy direct linear discriminant analysis; ELM, extreme learning machine.

**Table 8:** Classification accuracy of the model using naïve Bayes classifier with different preprocessing methods (%).

Feature Extraction	MSC	SNV	SG	MC	SG + MSC	SG + SNV	SG + MC
LDA	60.00	60.00	88.00	70.67	93.33	93.33	88.00
DLDA	46.67	46.67	90.67	61.33	90.67	90.67	90.67
FDLDA	52.00	52.00	89.33	66.67	92.00	92.00	89.33

Abbreviation: MSC: multiplying scattering correction; SNV: standard normal variables; SG: Savitzky–Golay; MC: mean centering; LDA: linear discriminant analysis; DLDA: direct linear discriminant analysis; FDLDA: fuzzy direct linear discriminant analysis.

## Data Availability

The original contributions presented in the study are included in the article, further inquiries can be directed to the corresponding authors.
